# An FAK Kinase/Scaffold Mode-Switch in Dormancy and Resistance

**DOI:** 10.3390/cancers18060995

**Published:** 2026-03-19

**Authors:** Changchang Sun, Qiuting Feng, Yiyang Zhao, Qihan Dong, Ling Bi

**Affiliations:** 1 Department of Medical Oncology, Shuguang Hospital, Shanghai University of Traditional Chinese Medicine, Shanghai 201203, China; reed_changc@163.com (C.S.); fengquiting@163.com (Q.F.); emma_zyy@126.com (Y.Z.); 2 School of Natural Sciences, Faculty of Science and Engineering, Macquarie University, Sydney, NSW 2109, Australia; 3 Department of Oncology, Yueyang Hospital of Integrated Traditional Chinese and Western Medicine, Shanghai University of Traditional Chinese Medicine, Shanghai 200437, China

**Keywords:** Focal adhesion kinase (FAK), Yes-associated protein (YAP), Cancer dormancy, Drug resistance, PROTACs

## Abstract

Late relapses are one of the most frustrating aspects of cancer treatment. They are frequently driven by dormant tumor cells and drug-tolerant persisters (DTPs) that survive therapy and later re-enter proliferation. Focal adhesion kinase (FAK) and the mechanosensitive transcriptional co-activators YAP/TAZ integrate extracellular matrix mechanics with intracellular stress signaling to coordinate survival, quiescence and reactivation. We propose that the key determinant is often not “FAK on/off”, but functional mode selection between (Mode I) kinase-dependent signaling bursts linked to adhesion remodeling and regrowth and (Mode II) kinase-independent scaffolding and non-canonical localization (including nuclear pools) that sustain a persistence architecture under stress. This Mode-Switch lens helps explain why ATP-competitive FAK inhibitors can suppress pY397-FAK-dependent outputs yet incompletely eradicate persister reservoirs and motivates strategies that remove FAK protein or disrupt persistence circuitry. We outline operational, pathology-compatible proxies for assigning dominant mode using composite readouts of pY397-FAK/total FAK, FAK localization, and YAP/TAZ/TEAD executor output. Finally, we discuss modality matching—kinase inhibition to suppress regrowth versus FAK degradation and/or YAP/TEAD blockade to dismantle persister reservoirs—as a testable framework for biomarker-stratified intervention in minimal residual disease.

## Simple Summary

Cancer recurrence often occurs because “dormant” tumor cells stop dividing to survive chemotherapy and reactivate later. This process involves Focal Adhesion Kinase (FAK) and its partner YAP. In this review, we propose that FAK acts as a functional switch: “Mode I” drives reactivation and cell growth, while “Mode II” acts as a structural shield that protects dormant cells. Most approved FAK inhibitors target only its kinase activity (Mode I) and do not dismantle the scaffold-dependent survival architecture (Mode II). We suggest that next-generation therapies capable of degrading the FAK protein entirely (PROTACs) may be required to remove this shield and eliminate persistent cancer cells.

## 1. Introduction

Cancer remains a major global health burden, and therapeutic resistance limits durable benefit across chemotherapy, targeted therapy and immunotherapy [1,2,3]. A key clinical challenge is that tumor cells can enter low-proliferative or quiescent states that evade therapies designed to eliminate dividing cells, persist for years, and subsequently reactivate to seed recurrence [4,5,6,7]. Drug-tolerant persisters (DTPs) represent a related phenotype in which a subpopulation survives acute therapy pressure in a reversible, stress-adapted state and later gives rise to resistant outgrowth [8,9].

This quiescent, dormant state is different from therapy-induced senescence. Quiescent dormancy refers to a reversible G_0_ state from which cancer cells can re-enter the cell cycle when permissive conditions are restored, without full commitment to the morphologic and genetic remodeling associated with senescence [10]. By contrast, therapy-induced senescence refers to a therapy-triggered durable senescent growth-arrest state with senescence-associated features [10,11,12]. Accordingly, TIS is not synonymous with G_0_/quiescence, and the focus in the present article applies specifically to reversible DTPs and dormant cells (G_0_ arrest).

Classical resistance mechanisms include altered drug targets, enhanced DNA repair, reduced apoptosis and increased drug efflux, but these do not fully explain late relapse when tumor burden appears controlled for long periods [13,14,15]. Dormancy/DTP biology offers a complementary explanation: survival without proliferation can preserve minimal residual disease until microenvironmental or therapy-driven changes enable re-expansion. Although dormancy and drug resistance are often discussed separately, they share core survival strategies. Both involve suppression of apoptosis, adaptive rewiring of signaling, and microenvironmental protection within specialized niches [16,17]. Accordingly, a framework that distinguishes mechanisms supporting persistence and survival from those driving reactivation and proliferative escape is needed to guide therapeutic design and biomarker selection.

The tumor microenvironment (TME) regulates dormancy entry, maintenance and exit through ECM composition, mechanical forces, hypoxia and immune-derived cues [18,19,20]. FAK is a non-receptor tyrosine kinase concentrated at focal adhesions, where it couples integrin engagement and mechanical inputs to downstream survival and motility pathways [21,22]. Meanwhile, YAP and TAZ are mechanosensitive transcriptional co-activators. Their nuclear activity reports cytoskeletal tension and stress signaling, thereby driving programs that support survival, plasticity and microenvironmental remodeling [23,24]. Recent work highlights the FAK–YAP axis as a driver of drug-tolerant persister cells and residual disease in lung and overran cancer [25,26]. FAK and YAP/TAZ are therefore well positioned to coordinate two seemingly opposing behaviors that define dormancy biology: maintaining viability during growth arrest and enabling efficient reactivation when conditions become permissive.

Thus, it is relevant to ask the question: how does the FAK–YAP/TAZ axis differentially affect persistence versus reactivation? We propose that the answer lies not in simple pathway activation levels, but in a functional Mode-Switch. We distinguish FAK kinase-dependent signaling bursts that can promote reactivation, proliferation and therapy resistance (Mode I), from kinase-independent scaffolding and nuclear/non-canonical functions (Mode II) that can maintain a persistence architecture and drug tolerance even when FAK catalytic activity is suppressed [27,28]. On this basis, we summarize how YAP/TAZ executes downstream transcriptional programs relevant to DTP survival and relapse, and we discuss translational implications for biomarkers and therapy modality selection.

Historically, FAK signaling has been viewed through a binary paradigm: catalytically active (pro-tumorigenic) versus inactive (bystander). The Mode-Switch model diverges fundamentally from this by positing that the catalytically ‘inactive’ state is an actively engaged scaffolding state critical for survival. While FAK’s dual kinase/scaffold functions have been elegantly dissected in vitro, the dynamic, reversible transition between these modes in vivo remains primarily a working hypothesis. This hypothesis is inferred from recent in vivo studies demonstrating FAK-dependent persistence in residual disease models where ATP-competitive kinase inhibitors fail to eradicate the tumor reservoir [26,27].

## 2. FAK Functional Mode Switching in Dormancy and Drug Tolerance

Focal adhesions are multi-protein assemblies that link integrins to the actin cytoskeleton and function as mechanochemical transducers. ECM stiffness and tensile load promote integrin clustering and talin unfolding, which recruits vinculin and strengthens the integrin–cytoskeleton linkage [29,30]. This reinforcement increases local tension and stabilizes nascent adhesions, creating a positive feedback loop between mechanics and signaling.

FAK is recruited to these adhesion sites and is activated by conformational changes and autophosphorylation at Tyr397. The resulting FAK–Src complex phosphorylates adhesome components such as paxillin at Tyr31/Tyr118, recruiting adaptors including Crk to coordinate adhesion maturation and turnover [21,31]. Downstream, RhoA–ROCK-mediated contractility amplifies focal adhesion signaling and modulates YAP/TAZ activity through tension-dependent mechanisms that may be partly Hippo-independent [23,32].

This mechanochemical circuitry is particularly relevant to dormancy and DTP biology because mechanical inputs are niche defining. Stiff, collagen-rich microenvironments tend to favor focal adhesion signaling and proliferative escape. In contrast, softer niches, reduced adhesion, or competing stress cues such as hypoxia and immune pressure can favor quiescence and stress adaptation [19,20]. We propose that FAK can support both behaviors via distinct functional modes. These modes are not mutually exclusive; rather, they represent dominant operating regimes along a spectrum shaped by adhesion dynamics, mechanical loading, and therapy stress.

### 2.1. Mode I—Kinase-Dependent Enzymatic Signaling with Reactivation-Associated Bursts

FAK is a modular protein with an N-terminal FERM domain, a central kinase domain and a C-terminal FAT domain. This allows for autoinhibition, focal-adhesion localization and regulated signaling output [33,34,35]. Upon integrin engagement and mechanical loading, FAK undergoes autophosphorylation at Tyr397, creating an SH2 docking site for Src-family kinases and initiating a phosphorylation cascade that couples to PI3K–AKT and RAS–MAPK signaling [36,37,38].

Mechanistically, this activation is driven by a conformational switch. Under high mechanical tension (e.g., stiff ECM), integrin clustering forces the autoinhibitory FERM domain away from the central kinase domain. This ‘open’ conformation exposes Tyr397 for rapid autophosphorylation, initiating the Mode I kinase burst [34].

In the context of dormancy, kinase-dependent signaling is most plausibly linked to reactivation because it amplifies proliferative and pro-survival programs. FAK–Src signaling can upregulate Cyclin D1 and suppress CDK inhibitors (p21/p27), favoring cell-cycle re-entry, while AKT/ERK signaling buffers therapy-induced stress and apoptosis [39,40,41]. Kinase activity also influences focal-adhesion turnover: phosphorylation-driven recruitment of adaptors at adhesion sites can accelerate adhesion disassembly and reassembly, supporting migration and niche exploration during relapse [27,42].

Kinase-dependent signaling is strongly implicated in therapy resistance. AKT/ERK pathways suppress pro-apoptotic regulators (e.g., Bad, Bim), stabilize anti-apoptotic proteins (Bcl-2 family) and inhibit stress-responsive transcription factors (FoxO), collectively sustaining survival during cytotoxic exposure [43,44]. In addition, in some tumor settings, FAK signaling has been linked to enhanced DNA repair capacity and radio/chemo-resistance, including in p53-altered tumors, although the precise mechanism and prevalence are context-dependent [45,46]. In ovarian cancer, FAK–YAP mechanosensing contributes to platinum resistance via nuclear YAP retention [25].

Kinase-dependent signaling also provides one route to YAP/TAZ activation. Mechanical stress promotes RhoA-driven tension and can trigger YAP nuclear accumulation in a manner not fully explained by canonical Hippo/LATS control, thereby connecting focal-adhesion signaling to TEAD-dependent transcription relevant to reactivation and resistance [23,47]. This is consistent with recent models where FAK–YAP sustains residual disease and persister phenotypes in lung cancer [26]. This linkage is central to understanding why FAK and YAP/TAZ often co-vary in aggressive or therapy-resistant states.

### 2.2. Mode II—Kinase-Independent Scaffolding and Persistence Architecture

A critical but often under-emphasized feature of FAK is that it functions as a scaffold at focal adhesions even when kinase activity is inhibited. When mechanical tension is lost—either through therapy-induced cytoskeletal collapse or within softer, hypoxic dormant niches—FAK reverts to a ‘closed’, autoinhibited conformation. Crucially, while this closed state masks the Tyr397 catalytic site (shutting down Mode I), the terminal FERM and FAT domains remain robustly anchored to paxillin, talin, and integrins. This conformational shift is the molecular trigger that transitions FAK into its Mode II scaffolding state [34,48]. Through interfaces mediated by the FERM and FAT domains, FAK coordinates integrins, paxillin, talin and other adhesome components to stabilize cell–ECM attachment and organize actin dynamics [48,49,50]. Mutations disrupting FAT-mediated binding to paxillin/talin impair focal-adhesion anchoring and directional migration, illustrating the functional importance of non-enzymatic interfaces [51,52].

In dormancy and DTP settings, such scaffolding can support a ‘persistence architecture’. For instance, the autonomous expression of the FAK scaffold domain (FRNK) has been shown to induce a dormant state where proliferation is arrested, yet survival pathways (e.g., Cas/Rac/JNK) remain active to prevent apoptosis [53]. Classical dormancy models highlight an ERK-to-p38 balance, where reduced integrin/adhesion signaling favors p38-dominant stress signaling and quiescence [54,55]. FAK scaffolding may permit a controlled intermediate: sufficient integrin-dependent survival input to avoid apoptosis, while stress signaling and growth arrest persist. This “survival without proliferation” phenotype aligns conceptually with DTP biology. However, much of the direct mechanistic evidence demonstrating that FAK scaffolding supports survival derives from in vitro adhesion and therapy-induced DTP models. Extrapolating these scaffolding survival mechanisms to classical, long-term clinical dormancy (e.g., solitary disseminated tumor cells residing in quiescent niches for years) remains speculative at present and requires definitive in vivo lineage-tracing validation.

Therapeutically, scaffold persistence means that catalytic shutdown is not equivalent to dismantling the adhesion-supported niche. ATP-competitive inhibitors suppress signaling bursts yet may leave non-catalytic survival circuitry intact, contributing to incomplete elimination of persister reservoirs and rapid rebound when drug pressure is relieved [27,56]. Recent PROTAC studies confirm superior disruption of both kinase and scaffold functions in ovarian and GI-NET models [56,57,58]. These considerations favor modalities that remove FAK protein or disrupt critical protein–protein interactions, especially when the clinical objective is eradication of minimal residual disease.

### 2.3. Nuclear FAK as a Compartmentalized Extension of Mode II Functions

FAK is not confined to focal adhesions. Under detachment, DNA damage, inflammatory cues, or therapy-associated rewiring, a fraction of FAK can redistribute and accumulate in the nucleus [59]. Within the Mode-Switch framework, this nuclear pool is best viewed as a compartmentalized extension of Mode II, where kinase-independent or weakly kinase-coupled functions can become dominant [45].

Because nuclear FAK lacks the spatial coupling needed for adhesion-based catalytic bursts, its reported functions are most plausibly mediated through protein–protein interactions (e.g., FERM-dependent scaffolding) that organize nuclear complexes and modulate transcriptional control nodes [28,60,61]. Functionally, this provides a mechanism by which cells can maintain stress-adaptive programs during persistence while remaining poised for later reactivation: nuclear scaffolding can support survival and transcriptional plasticity without requiring sustained Mode I kinase flux.

Operationally, nuclear Mode II should be assigned only when nuclear FAK is demonstrable (IHC/IF nuclear score or % nuclear-positive tumor cells), together with evidence of low/uncoupled kinase flux (pY397-FAK/total FAK and downstream pAKT/pERK) and a stress-adapted transcriptional context. This composite definition keeps nuclear FAK inside the Mode II category without over-claiming universal function.

### 2.4. Integrating Modes Across the Dormancy Timeline

Taken together, the two modes provide a parsimonious framework for understanding the dual role of the FAK axis in dormancy biology, but they should be interpreted as dominant operating regimes rather than mutually exclusive labels. In general, Mode I (kinase-dependent bursts) becomes prominent during escape, reactivation, and aggressive outgrowth when proliferative cues and adhesion remodeling are engaged, whereas Mode II (kinase-independent scaffolding/nuclear persistence architecture) is favored under dormancy or DTP stress where survival and mechanical continuity are prioritized. Importantly, cells can exhibit mixed features and switch dynamically as ECM composition, therapy stress, and microenvironmental inputs evolve; therefore, mode assignment should be treated as state-dependent rather than a fixed tumor property.

The Mode I–Mode II boundary is defined operationally using a composite readout (Table 1). Mode I (“kinase-burst”) is defined by high catalytic flux: elevated pY397-FAK (preferably normalized to total FAK), robust Src-coupled signaling and downstream pAKT/pERK, typically accompanied by adhesion remodeling and high mechanotransduction output (often reflected by higher-amplitude nuclear YAP/TAZ). Mode II (“scaffold-dominant”) is defined by uncoupling between catalytic flux and persistence architecture: low or intermittent pY397-FAK despite sustained FAK residency within adhesions (high FAK–paxillin/talin/vinculin association; adhesion-enriched punctate localization) and/or non-canonical localization such as a nuclear FAK pool. In this regime, YAP/TAZ output may be lower in amplitude yet still biologically meaningful (e.g., survival-biased transcription rather than overt proliferation).

**Figure 1:**
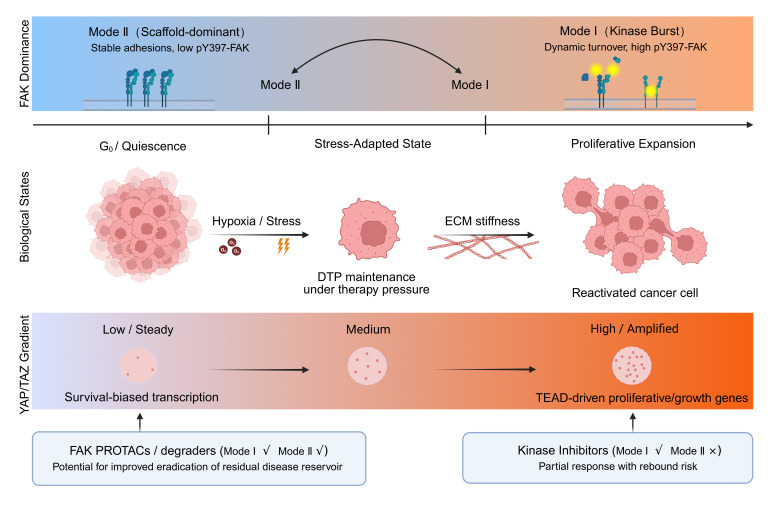
Dormancy/DTP state machine mapped to FAK operating modes and YAP/TAZ executor output. Conceptual schematic of a dormancy/DTP timeline in which FAK signaling is described by two dominant regimes: Mode II (kinase-independent scaffolding depicted by stable, blue closed-conformation FAK molecules, and/or non-canonical localization, including nuclear pools) is enriched during entry into dormancy and persistence under stress, whereas Mode I (kinase-dependent signaling with high pY397-FAK, depicted by FAK molecules with yellow phosphorylation bursts) is associated with reactivation and escape. The gradient illustrates corresponding shifts in YAP/TAZ/TEAD transcriptional output from survival-biased programs toward proliferative programs. In this framework, kinase inhibitors can suppress Mode I burst signaling yet may spare Mode II persistence architecture, while FAK degraders can remove FAK protein and thereby disrupt both kinase-dependent and scaffold-dependent functions. Mode assignment is intended as a state-dependent continuum and may shift with ECM context and therapy pressure. (The "√" and "×" in the image indicate whether the drug strategy is effective for the two distinct FAK signaling modes (Mode I and Mode II)).

This operational framing clarifies why catalytic inhibition is not equivalent to dismantling persistence circuitry: kinase inhibitors can suppress Mode I bursts while leaving Mode II architecture intact. Crucially, the “trigger” for mode switching is not limited to inherent ECM stiffness; it may be actively induced by clinical intervention. For example, therapy-driven remodeling of the stroma, altered adhesion ligand availability, or treatment-associated stress responses can shift surviving cells away from a proliferative kinase-burst state and into a scaffold-dominant persistence regime. Recognizing therapy as a selective pressure that can shaft the dominant FAK operating mode is therefore essential for rational timing and modality selection—particularly when considering degraders that remove FAK protein and can disrupt both adhesion and nuclear scaffold functions (Figure 1). Importantly, FAK mode dominance is highly context- and tumor-type-dependent, varying with ECM composition, mechanical cues, therapeutic pressure, and intrinsic cellular wiring across different malignancies. Accordingly, Table 1 summarizes practical proxies that jointly report kinase flux, localization/scaffolding, and YAP/TAZ executor output to support robust mode assignment.

## 3. YAP/TAZ as the Execution Layer in Persistence Biology

YAP and TAZ are transcriptional co-activators lacking DNA-binding domains. Instead, they cooperate with transcription factors such as TEAD to regulate gene programs controlling proliferation, survival and tissue remodeling [24,62]. Canonical Hippo signaling restrains YAP/TAZ via inhibitory phosphorylation and cytoplasmic retention, whereas stiff matrices, focal adhesion signaling and RhoA-driven contractility favor nuclear accumulation and transcriptional activity [63,64]. Because focal adhesions and actomyosin tension sit upstream of YAP/TAZ, perturbations that reshape adhesion mechanics, including via FAK, can rapidly reconfigure YAP/TAZ output [23].

### 3.1. Control of Nuclear Amplitude and Dynamics

The primary mechanism of YAP/TAZ regulation is control of their nuclear or cytoplasmic localization. Mode I (kinase-burst) signaling is a potent driver of nuclear accumulation. FAK-Src and RhoA/ROCK activity, hallmarks of Mode I, generate cytoskeletal tension that inhibits the Hippo kinase LATS1/2, the main cytoplasmic sequester of YAP/TAZ [63,64]. This establishes a clear link where high Mode I activity leads to tension, which leads to LATS inhibition and finally high nuclear YAP/TAZ. Conversely, Mode II (scaffold-persistence) states, often associated with softer niches or stable adhesions, may maintain lower, yet functionally significant, levels of nuclear YAP/TAZ. This could occur through partial or localized inhibition of Hippo signaling, or through Hippo-independent mechanisms that permit a survival-optimized nuclear pool without triggering full proliferative transcription. Thus, we predict that Mode I should correlate with maximal nuclear YAP/TAZ amplitude, while Mode II may sustain a lower, steadier nuclear presence.

In Mode II, the scaffolding shield likely regulates YAP indirectly in the cytoplasm. By maintaining basal tethering to paxillin and vinculin at stable adhesions, the Mode II scaffold preserves residual actin cytoskeletal architecture. This provides just enough mechanical continuity to prevent complete YAP degradation, maintaining a ‘Low/Steady’ survival-biased pool (as visually depicted in the YAP/TAZ Gradient of Figure 1), without generating the high actomyosin tension required for Mode I proliferative bursts.

### 3.2. Influence on the Post-Translational Landscape

Beyond localization, the functional output of nuclear YAP/TAZ is modulated by phosphorylation, acetylation, and other modifications [24,65]. Mode I signaling may directly imprint a “proliferative” mark on YAP/TAZ. For instance, adhesion-associated kinase networks that are frequently engaged during Mode I signaling can introduce post-translational modifications to YAP/TAZ that favor stability and growth-promoting transcription [66,67,68].

However, such modification signatures are context-dependent and should not be treated as universal direct readouts of FAK catalytic activity. Consequently, Mode II-dominant persistence does not necessarily imply absence of these events; rather, it may shift the balance toward a modification landscape shaped by stress-activated kinases such as p38 or metabolic sensors, potentially recruiting different transcriptional co-factors. Accordingly, phospho-signatures should be interpreted alongside mode proxies including pY397-FAK and FAK localization and validated in the relevant dormancy/DTP models.

### 3.3. Nuclear FAK Scaffolding as a Direct Transcriptional Modulator

As a Mode II component, nuclear FAK functions as a scaffold. While direct physical interaction between nuclear FAK and YAP/TAZ complexes has not been fully mapped, a logical hypothesis emerges. Nuclear FAK could architect chromatin environments or recruit co-regulators that favor the “Adaptive/Survival” transcriptional program. Evidence supporting this model includes that nuclear FAK associates with transcriptional regulators and chromatin in some systems and the requirement of YAP/TAZ for interaction with diverse chromatin regulators for context-specific transcription [69]. It is therefore plausible that nuclear FAK, via its scaffolding role, helps assemble or stabilize a transcriptional complex on chromatin that biases YAP/TAZ toward survival gene expression at the BCL-xL locus rather than proliferative genes [70,71].

Taken together, we propose that Mode I drives high-amplitude, proliferation-biased YAP/TAZ activity through tension-mediated Hippo inhibition and potentially Src-dependent YAP phosphorylation, whereas Mode II sustains lower-amplitude, survival-biased YAP/TAZ output through stable adhesion scaffolds and possibly nuclear FAK-assisted transcriptional complexes.

However, direct physical evidence linking nuclear FAK to YAP/TAZ transcriptional complexes remains a critical gap in the field. To validate this hypothesis, future studies must employ endogenous co-immunoprecipitation (co-IP) to confirm whether direct or indirect protein–protein interactions exist between nuclear FAK and the YAP/TAZ/TEAD complex. Furthermore, chromatin immunoprecipitation sequencing (ChIP-seq) is required to definitively map whether FAK and YAP co-occupy specific survival-associated promoters (such as BCL2L1/BCL-xL) under therapy-induced stress, thereby proving its direct functional role as a transcriptional scaffold.

## 4. Translational Implications: Biomarker Logic and Modality Matching

Recognizing that FAK and YAP/TAZ coordinate survival and regrowth through these distinct operating modes fundamentally alters our clinical perspective. It suggests that treating the pathway as a simple binary switch is insufficient. Instead, we must translate this biological complexity into precise therapeutic strategies. This shift requires us to move beyond generic expression levels and identify specific biomarkers that reveal which functional mode is dominant, thereby guiding the selection of the most effective intervention.

### 4.1. Biomarkers Should Capture State, FAK Mode and YAP/TAZ Executor

The Mode-Switch framework implies that single-node expression measurements are insufficient. In addition to a dormancy/DTP state readout, clinically useful stratification should report FAK mode using (i) pY397-FAK intensity normalized to total FAK as a proxy for kinase flux and (ii) subcellular localization (focal-adhesion-associated versus nuclear/cytoplasmic FAK). A scaffold-dominant regime is suggested by low or uncoupled pY397-FAK despite persistent adhesome residency (e.g., high FAK–paxillin/talin association) and/or a measurable nuclear FAK pool. YAP/TAZ executor output should be co-reported (e.g., YAP/TAZ nuclear localization and/or TEAD target signatures such as CTGF/CYR61) [72,73,74]. Importantly, these markers should be interpreted in a compartment-aware manner (tumor vs. stromal/endothelial) and, where possible, using spatially resolved scoring to avoid conflating biologically distinct sources of signal.

To operationalize Mode II (scaffold/nuclear) contributions without relying on non-clinical kinetics assays, localization can be converted into a pathology-compatible score. For example, using IHC or multiplex IF with digital analysis, nuclear FAK can be quantified as an H-score (intensity × % positive nuclei) or as a % nuclear-positive tumor cells, while adhesion-enriched FAK/pY397-FAK can be quantified as peripheral/punctate staining co-localizing with focal-adhesion markers (e.g., paxillin/vinculin) and summarized as puncta density or adhesion-area fraction [75]. A simple composite such as a nuclear-FAK/adhesion-FAK or nuclear-FAK/pY397-FAK ratio may serve as a pragmatic proxy of scaffold dominance.

While theoretically robust, simultaneously quantifying these multiple spatial and intensity-based metrics presents significant practical challenges for routine clinical pathology. Standard immunohistochemistry (IHC) is often insufficient for resolving nuanced adhesion-versus-cytosolic distribution. Transitioning this framework to the clinic will likely require the standardization of multiplex immunofluorescence (mIF) panels coupled with automated, AI-driven digital pathology algorithms to reproducibly calculate composite scores across heterogeneous tumor sections. Alternatively, future research should explore whether downstream, easily measurable secreted surrogate markers of the Mode II/YAP survival state exist, which could bypass the need for complex spatial profiling altogether.

Dynamic and functional readouts—such as FAK turnover at adhesions (e.g., FRAP in engineered or live-cell systems) or ex vivo differential sensitivity to ATP-competitive inhibitors versus degraders—are best positioned as research-grade or early-trial companion assays, rather than routine clinical biomarkers. Their value is to validate mode assignment, quantify mode switching under therapy, and refine modality matching in model systems, patient-derived organoids, or fresh biopsy platforms, with the longer-term goal of identifying static surrogate markers that can be deployed in standard diagnostic pipelines.

Ultimately, the clinical utility of these composite biomarkers lies in patient selection: identifying patients with a baseline ‘Mode II-dominant’ profile who are unlikely to achieve durable responses with standard kinase inhibitors and should instead be prioritized for degrader-based or combination trials.

### 4.2. Why ATP-Competitive FAK Inhibitors Can Suppress Regrowth Yet Spare Persister Reservoirs

Multiple ATP-competitive FAK inhibitors suppress Tyr397 autophosphorylation and downstream signaling reducing invasion and tumor growth in preclinical models [27]. Representative agents include defactinib (VS-6063), PF-562,271 and GSK2256098, which broadly suppress kinase output and have been evaluated across solid tumors [76,77,78,79] (Table 2).

It is crucial to emphasize that these agents exclusively target Mode I functions. They successfully block ATP binding and subsequent catalytic bursts but are structurally incapable of dismantling the Mode II protein–protein scaffolding interfaces that tether the cell to the ECM. Thus, clinical efficacy has often been modest as monotherapy.

This is consistent with two mechanistic limitations. First, compensatory pathway activation (e.g., MAPK/STAT3/PI3K feedback) can blunt the impact of catalytic inhibition [80,81]. Notably, these compensatory signals may be specifically sustained by the remaining Mode II scaffold [57,58], which continues to anchor the cell to the ECM and provide a structural foundation for alternative growth factor receptor signaling. Second, kinase-independent scaffolding and nuclear/non-canonical functions can maintain persistence architecture and stress-adaptive programs despite catalytic suppression [27]. By leaving the physical adhesome and nuclear FERM-mediated interactions intact, ATP-competitive inhibitors may inadvertently stabilize the Mode II state, potentially stabilizing a drug-tolerant, scaffold-dominant survival configuration rather than inducing apoptosis [27,56]. Thus, catalytic inhibition may be most effective when the dominant biology is kinase-burst-driven such as during reactivation-associated regrowth, whereas scaffold-dominant persistence states may require protein removal or combination approaches.

### 4.3. Modality-Aware Strategies: Degraders, YAP/TAZ Blockade, and Combinations

FAK degraders (e.g., PROTAC approaches) are conceptually attractive because they remove both catalytic and scaffolding functions. This potentially overcomes a key limitation of kinase inhibitors [56,57,58]. Consistent with this logic, a reported FAK-targeting PROTAC outperformed a parent inhibitor in preclinical ovarian cancer models and disrupted both signaling and protein–protein interaction functions [57]. While FAK-targeting PROTACs have demonstrated preclinical superiority over kinase inhibitors in disrupting both catalytic and scaffolding functions, their clinical translation remains in early stages, with no FAK-specific PROTACs currently reported in advanced (phase II/III) trials.

Genetic evidence indicates that FAK is essential during development: FAK loss or catalytic inactivation causes embryonic lethality in mice at gastrulation [82]. In contrast, inducible/tissue-restricted FAK deletion in adult mice can show normal baseline tissue structure and function in some contexts such as cardiomyocyte [83]. In early phase I studies of pharmacologic FAK targeting in advanced solid tumors, commonly reported adverse events included nausea, fatigue, vomiting, diarrhea and headache, and reversible unconjugated hyperbilirubinemia was observed as a dose-limiting toxicity in some cohorts [84].

Targeting the transcriptional executor provides a complementary axis. Agents that disrupt YAP–TEAD transcriptional activity like verteporfin in preclinical contexts illustrate the feasibility of attenuating YAP-driven programs that support DTP survival and relapse [85,86]. Mode-aware combinations therefore have a clear rationale: (i) FAK degrader plus YAP/TEAD blockade to target both persistence architecture and execution; (ii) kinase inhibition plus YAP/TEAD blockade to suppress reactivation bursts and downstream transcription; or (iii) FAK-targeted agents plus ECM/adhesion modulators to lower mechanical inputs that drive the circuit [19,87].

### 4.4. Scenario Logic: Aligning the Intervention with the Clinical Goal

A practical advantage of the Mode-Switch framework is that it clarifies which biological objective is being pursued. If the objective is to suppress regrowth or prevent reactivation-associated expansion, kinase inhibition may be sufficient in biomarker-defined settings where pY397-FAK and YAP/TAZ nuclear activity are high. If the objective is to eliminate persister reservoirs and reduce late relapse risk, approaches that dismantle persistence architecture (FAK degradation, adhesion/ECM disruption) and/or block YAP/TEAD execution are more logically aligned.

This suggests a stepwise deployment logic: (i) use state and mode biomarkers to identify whether the dominant vulnerability is kinase-burst or scaffold persistence; (ii) choose modality accordingly (inhibitor vs. degrader ± YAP blockade); and (iii) incorporate microenvironmental context including stiffness, hypoxia, and immune niche when selecting combinations. Such mode-aware deployment may help rationalize heterogeneous trial results and guide future biomarker-stratified studies.

### 4.5. Practical Challenges: Selectivity, Scaffolding Druggability, and Adaptive Escape

Several practical issues shape translation. First, kinase inhibitors can have off-target effects due to kinase-domain homology with related kinases (e.g., PYK2), and toxicity can limit dose intensity, motivating more selective or allosteric approaches [27,77]. Second, scaffolding functions are difficult to inhibit with traditional small molecules because protein–protein interfaces in FERM/FAT domains often lack deep binding pockets; this is a major reason degraders and interaction-focused strategies are attractive. Third, pathway plasticity enables adaptive escape. A therapy that successfully targets Mode I might select for or induce a shift to Mode II, growth factor receptor signaling, or alternative transcriptional programs. These realities argue for the need for longitudinal biomarker monitoring and combined therapies with clear mechanistic rationale. Thus, the lack of clinical agents capable of selectively targeting the scaffold domain without broad toxicity risks remains a major limitation of current FAK-targeted therapies.

Despite the conceptual appeal of FAK-directed PROTACs to overcome scaffolding-mediated resistance and eliminate persistence architecture, their clinical translation faces substantial pharmaceutical and safety hurdles that must be carefully addressed. PROTACs are inherently large, heterobifunctional molecules that often violate classical drug-likeness rules (e.g., Lipinski’s Rule of Five), leading to suboptimal pharmacokinetics and poor oral bioavailability. Achieving sufficient drug penetration into the dense, desmoplastic tumor microenvironments that often harbor DTPs poses another major physical barrier. Finally, the degradation kinetics must be finely tuned to avoid the ‘hook effect’—where excessively high drug concentrations saturate both the target and the E3 ligase independently, thereby disrupting the ternary complex formation required for FAK degradation.

## 5. Conclusions and Outlook

### 5.1. Summary

FAK–YAP/TAZ signaling serves as a central conduit through which ECM mechanics and stress cues shape survival, dormancy and therapy resistance. By explicitly separating kinase-dependent signaling bursts from kinase-independent scaffolding and nuclear/non-canonical functions, a Mode-Switch lens clarifies how the same pathway can support both persistence and reactivation [21,23,27]. This distinction explains the clinical observation that FAK kinase inhibition is rarely synonymous with complete pathway shutdown.

While the FAK Mode-Switch model offers a mechanistic explanation for persistence and reactivation in dormancy/DTP biology, much of the supporting evidence derives from preclinical models (e.g., cell lines, organoids, and xenograft studies), with limited direct validation in patient-derived dormant or residual disease states. Nevertheless, the framework generates clear, testable hypotheses that are now amenable to investigation in patient-derived organoid models and neoadjuvant window-of-opportunity trials. Operational mode assignment currently relies on static proxies (e.g., pY397-FAK levels, subcellular localization, YAP/TAZ nuclear scoring), which may not fully capture dynamic switching under therapeutic pressure or heterogeneous tumor microenvironments. Longitudinal, real-time biomarkers are needed to track mode transitions clinically. Furthermore, although FAK PROTACs show superior preclinical efficacy in targeting both modes, their translation faces hurdles including potential on-target toxicity from complete FAK depletion and the absence of FAK-specific degraders in advanced (phase II/III) clinical trials as of 2025–2026. These gaps underscore the importance of rigorous validation in patient cohorts and adaptive trial designs.

### 5.2. Outlook: Key Priorities

The priority for future research is to move beyond static snapshots and capture the dynamic nature of this mode switching. Dormancy and drug-tolerance models should routinely quantify functional proxies such as pY397-FAK levels, subcellular localization, and adhesome dynamics alongside YAP/TAZ output. This is essential to determine which mode dominates under specific therapeutic and microenvironmental conditions. A critical focus is how initial therapeutic pressure (including chemotherapy or targeted therapy) can select for, or induce, a shift from Mode I features toward a Mode II persistence regime, as inferred by longitudinal changes in pY397-FAK/total FAK, FAK localization, adhesome proxies, and YAP/TAZ output. Furthermore, we need to resolve how the FAK scaffold sustains compensatory signaling loops even when its kinase activity is blocked. Understanding this interplay will be essential to transition from the passive inhibition of regrowth to the active eradication of the minimal residual disease reservoir.

## Figures and Tables

**Table 1:** FAK mode in dormancy/DTPs: operational signatures and therapeutic predictions.

FAK Mode	Likely Position on Dormancy Timeline	Operational Signature (Minimum Composite)	Therapeutic Implication (Prediction)
Mode I: kinase-dependent signaling	More prominent during reactivation/escape and aggressive outgrowth; can also appear as transient bursts under therapy.	High kinase flux: ↑ pY397-FAK/total FAK; Src-network engagement; ↑ pAKT and/or ↑ pERK. Often higher-amplitude nuclear YAP/TAZ (context-dependent).	ATP-competitive kinase inhibitors can blunt bursts and suppress regrowth, but may not eradicate persisters if Mode II architecture persists.
Mode II-A: adhesion scaffold–dominant persistence architecture	During dormancy/DTP maintenance; often selected/enriched by therapy pressure and hostile microenvironment cues.	Uncoupled catalytic flux: low/intermittent pY397-FAK despite persistent adhesion residency (FAK co-localization with paxillin/talin/vinculin; adhesion puncta density/adhesion-area fraction; high FAK–adhesome association). Survival wiring may persist without strong proliferative signaling.	Kinase inhibitors may spare scaffold function. FAK degraders/PROTACs (or PPI-disrupting strategies) are predicted to better dismantle persistence circuitry; combinations may be required depending on executor outputs.
Mode II-B: nuclear scaffold–dominant (context-dependent)	Stress-adapted persistence states; may also prime later reactivation (model- and context-dependent).	Non-canonical localization: measurable nuclear FAK pool (IHC/IF nuclear score; % nuclear-positive tumor cells), often with stress-adaptive transcriptional programs; can coexist with adhesion scaffold features.	Strengthens rationale for protein removal approaches (degraders) when nuclear scaffolding is prominent; kinase inhibition alone is unlikely to disable nuclear scaffold functions.
Hybrid/transition state	Entry into dormancy, early DTP adaptation, pre-reactivation, or shifting ECM/therapy contexts	Mixed features: concurrent evidence of kinase flux (↑ pY397-FAK and downstream pAKT/pERK) and scaffold dominance (persistent adhesome and/or nuclear localization).	Supports mode-aware combinations and longitudinal reassessment; avoids forced binary labeling when biology is mixed.

Footnote: Mode assignment should be compartment-aware (tumor vs. stromal/endothelial) and localization-aware (adhesion vs. cytosolic vs. nuclear), because identical markers can report different biology depending on cellular source and subcellular distribution. The ↑ symbol indicates an increase.

**Table 2:** Clinical Status of Representative FAK Inhibitors.

Agent	Mechanism	Clinical Status	Limitations
Defactinib (VS-6063)	ATP-competitive kinase inhibitor (Mode I target)	Phase II/III	Modest monotherapy efficacy; currently evaluated in combinations (e.g., with RAF/MEK inhibitors) for ovarian and lung cancers.
PF-562,271	ATP-competitive kinase inhibitor (Mode I target)	Phase I	Clinical development discontinued due to non-linear pharmacokinetics and limited single-agent activity.
GSK2256098	ATP-competitive kinase inhibitor (Mode I target)	Phase I/II	Tolerable, but failed to show robust objective response rates as a monotherapy in advanced solid tumors.

## Data Availability

No new data were created or analyzed in this study. Data sharing is not applicable to this article.

## Abbreviations

The following abbreviations are used in this manuscript:

BCL-2	B-Cell Leukemia/Lymphoma 2
BCL-xL	B-Cell Lymphoma-extra Large
CTGF	Connective Tissue Growth Factor
CYR61	Cysteine-Rich Angiogenic Inducer 61
DLC1	Deleted in Liver Cancer 1
DTP	Drug-Tolerant Persister
ECM	Extracellular Matrix
EGFR-TKI	Epidermal Growth Factor Receptor Tyrosine Kinase Inhibitor
ER	Endoplasmic Reticulum
ERK	Extracellular Signal-Regulated Kinase
FAK	Focal Adhesion Kinase
FAT	Focal Adhesion Targeting
FERM	4.1, Ezrin, Radixin, Moesin
HCC	Hepatocellular Carcinoma
HIF-1α	Hypoxia-Inducible Factor 1-alpha
HPV	Human Papillomavirus
ICI	Immune Checkpoint Inhibitor
IGF-1R	Insulin-like Growth Factor 1 Receptor
LATS1/2	Large Tumor Suppressor Kinase 1/2
MAPK	Mitogen-Activated Protein Kinase
NSCLC	Non-Small Cell Lung Cancer
PDAC	Pancreatic Ductal Adenocarcinoma
PD-L1	Programmed Death-Ligand 1
PI3K	Phosphoinositide 3-Kinase
PROTAC	Proteolysis Targeting Chimera
STAT3	Signal Transducer and Activator of Transcription 3
TAZ	Transcriptional Co-activator with PDZ-binding Motif
TEAD	TEA Domain Transcription Factor
TGF-β	Transforming Growth Factor Beta
TME	Tumor Microenvironment
TNBC	Triple-Negative Breast Cancer
YAP	Yes-Associated Protein
